# Physicians’ Perception of Oral Nutritional Supplement Acceptance and Tolerability in Malnourished Outpatients: PerceptiONS Study

**DOI:** 10.3390/nu15051219

**Published:** 2023-02-28

**Authors:** P. B. Pedrianes-Martin, C. Dassen-de-Monzo, J. M. Guardia-Baena, M. Riestra-Fernández, C. Salom-Vendrell, PerceptiONS Group, A. Calvo-Barbero, L. Lizán-Tudela

**Affiliations:** 1 Section of Endocrinology and Nutrition, University Hospital of Gran Canaria Doctor Negrín, 35010 Las Palmas de Gran Canaria, Spain; 2 Service of Endocrinology and Nutrition, University Hospital Fundación Jiménez Díaz, 28040 Madrid, Spain; 3 Service of Endocrinology and Nutrition, University Hospital Virgen de las Nieves, 18014 Granada, Spain; 4 Endocrinology and Nutrition Department, University Hospital of Cabueñes, 33394 Gijón, Spain; 5 Endocrinology, Nutrition, Diabetes and Obesity Group, Health Research Institute of the Principality of Asturias (ISPA), 33011 Oviedo, Spain; 6 Service of Endocrinology, University Hospital Doctor Peset, 46017 Valencia, Spain; 7 Health Economics & Outcomes Research, Outcomes’10 S.L, 12071 Castellón de la Plana, Spain; 8 Medicine Department, Jaume I University, 12071 Castellón de la Plana, Spain

**Keywords:** acceptance, adherence, malnourished, malnutrition, non-hospitalized, oral nutritional supplements, outpatients, satisfaction, tolerability

## Abstract

Malnutrition is a common condition associated with various pathologies such as infections, neoplasms and digestive system disorders. Patients can be managed using different strategies, which include dietary modifications or oral nutritional supplements (ONS). It is important to promote good ONS adherence in order to attain clinical efficacy and cost-effectiveness. Several factors (amount, type, duration and tolerability) may have an impact on ONS adherence. PerceptiONS is a descriptive, cross-sectional observational study based on an ad hoc electronic survey designed to explore physicians’ perception of malnourished outpatients prescribed ONS. The survey considered adherence, acceptance/satisfaction, tolerability and benefits within the context of Spain’s healthcare system. The perceptions of 548 physicians regarding the experience of 2516 patients were analyzed. From the physicians’ perspective, 57.11% of patients adhered to over 75% of the prescribed ONS. The organoleptic properties of ONS represented the aspect with the most positive impact on adherence, with smell (43.72%) ranking as the top characteristic. In general, patients were satisfied (90.10%) with the ONS, with their related benefits (88.51%) and their organoleptic properties (90.42%), and accepted ONS in their daily diet (88.63%). ONS improved patients’ general condition (87.04%), quality of life (QoL) (81.96%) and vitality/energy (81.28%). Physicians would prescribe the same ONS again in 96.4% of the cases.

## 1. Introduction

Nutrition plays a critical role in maintaining the health and wellbeing of individuals and is also an essential component of the healthcare delivery system [1]. The nutritional requirements of healthy individuals depend on several factors, including age, sex and activity [1]. An imbalance in nutritional intake leads to malnutrition [1], which arises from deficient energy and protein intake, and commonly occurs among community-dwelling individuals in developed countries [2]. Maintaining an adequate nutritional status as well as sufficient nutrient intake is key to health and quality of life (QoL) and is, thus, a prerequisite for wellbeing at older ages and a modulator of healthy aging, as defined by the World Health Organization (WHO) [3]. Disease-related malnutrition has detrimental clinical effects, increasing mortality and morbidity and affecting the QoL, as well as having a high economic impact on the healthcare system [4].

Malnutrition is a common condition associated with a variety of pathologies. These include acute diseases such as infections, surgery and chronic conditions such as neoplasms or gastrointestinal disease.

Several conditions such as anorexia, nausea, increased catabolism and related digestive system disorders (dysphagia or malabsorption) may lead to malnutrition [5], and it is well established that malnutrition is associated with increased mortality in both acute and chronic disease [6]. Malnutrition prevalence is difficult to assess due to its different etiologies and the existence of more than one diagnostic tool [7]. The prevalence of hospital malnutrition can reach 30% according to the New Global Malnutrition Definition (GLIM) criteria and increases in the elderly [2,8]. Malnutrition is a frequent complication of a primary disease and should, thus, be adequately addressed due to the fact that the nutritional status of individuals affects their clinical outcomes [1]. Data on the prevalence of malnutrition and nutritional risk in the elderly across different healthcare settings show a wide range of malnutrition, oscillating between 8.5% in the community setting and approximately 30% in rehabilitation and subacute care conditions [9].

The elderly represent a large percentage of the population affected by malnutrition; however, other groups, such as cancer patients and patients with chronic conditions, are also at risk of becoming malnourished. Thus, malnutrition has been reported in 25–70% of cancer patients [7] and in 20% of the population with chronic diseases such as chronic obstructive pulmonary disease [10]. In this context, malnutrition is considered to be an important prognostic factor in cancer patients [11]. In this respect, cancer patients with malnutrition may suffer a negative clinical outcome, including premature death, with research reporting that around 20–30% of patients with malignancies die due to tumor-related malnutrition [11].

The diagnosis and assessment of malnutrition are still challenging and, therefore, it is often undiagnosed and untreated, despite the large population at risk and the associated burden [12]. Currently, more than 70 nutritional screening tools have been developed for use in hospitals to facilitate the evaluation of patients’ nutritional status and predict poor clinical outcomes related to malnutrition [13]; however, practical and implementable clinical screening tools to support diagnosis are lacking [13].

The high-prevalence and adverse consequences of malnutrition call for the early identification of malnourished subjects and prompt and effective treatment [14]. The majority of patients with or at risk of malnutrition can be managed following different strategies, including: (1) dietary counseling as indicated in clinical guidelines, (2) increased dietary food consumption and (3) using convenient oral nutritional supplements (ONS) [14].

ONS are sterile liquids, semi-solids or powders, which provide macro and micronutrients, and are designed for oral consumption; thus, taste and format are important to improve patient adherence [4]. ONS utilization has been consistently associated with both patient-reported and clinical benefits, such as an increase in body weight (weighted mean difference 2.25 kg; 95% CI 1.7–2.7) and muscle strength, functional improvements (walking distances and activities of daily living), immune function and QoL [14]. Furthermore, several clinical trials conducted in hospital and community settings in a malnourished population receiving ONS showed a reduction in complications associated with malnutrition (RR 0.44; 95% CI 0.3–0.6), and lower mortality rates in comparison with routine care (OR 0.7; 95% CI 0.5–0.9) [14]. In addition to evidence supporting the benefits of ONS provided by clinical trials and observational studies, ONS use is also endorsed by the main scientific societies working in this ambit, such as the American Society for Parenteral and Enteral Nutrition (ASPEN) and the European Society for Clinical Nutrition and Metabolism (ESPEN), as an early approach to tackle malnutrition when enteral feeding is not contraindicated [15,16,17]. Moreover, ONS is also recommended in the perioperative period [18] for cancer patients [18] and for patients with liver [19] and kidney disease [20], to mention but a few.

ONS are clinically effective in managing malnutrition if prescribed to malnourished patients or those at risk of malnutrition [15,16,17]. A recent systematic review indicated that ONS combined with dietary counseling in the elderly at risk of malnutrition is the most effective intervention, leading to increased dietary intake and weight [21]. These authors also explored whether the effects of intervention were influenced by study characteristics or participants’ traits. Their results indicated that females as well as older and more underweight participants were more likely to respond to the intervention by increasing their energy intake [21].

Good adherence to ONS is important to achieve both clinical efficacy and cost-effectiveness [4]. The literature suggests adherence is variable, possibly due to differences in study design [4]. Adherence to ONS is usually hampered by individual flavor perception, which may limit oral intake [4]. Therefore, to improve adherence to ONS, recent efforts have aimed to develop novel ONS with flavors, texture and compositions according to patients’ preferences [22]. ONS tolerability is another barrier to adherence since the development of gastrointestinal symptoms may lead to their discontinuation [23]. A recent systematic review found the adherence to enteral nutrition of hospitalized patients to be >80%, but this dropped to 50% in non-hospitalized patients [23]. ONS adherence may also depend on the amount, type and duration of supplementation [23]. Low adherence is not limited to ONS, and patients’ adherence can be challenging even for persons with home enteral nutrition, where up to 10% of users fail to reach 80% of the prescribed total dose [24].

ONS provide a nutritional support option that is widely used and recommended in various clinical situations associated with disease-related malnutrition. This study aims to explore physicians’ perception of patients’ adherence, acceptance/satisfaction and benefits in malnourished outpatients receiving ONS in the Spanish public healthcare system.

## 2. Materials and Methods

PerceptiONS is a descriptive, cross-sectional observational study based on an ad hoc electronic survey designed to explore physicians’ perception of malnourished outpatients’ adherence, acceptance/satisfaction, tolerance and benefits of receiving ONS.

The study was led by a steering committee, composed of five Spanish endocrinological experts in malnutrition management. All the members of the steering committee were working in a hospital within the Spanish public healthcare system. They were selected based on their extensive experience in malnutrition management (supported by their participation in studies and/or publications in this field) and their willingness to participate in the study.

### 2.1. Study Participants

The electronic survey addressed physicians working in Spain’s public healthcare system with experience in managing malnourished adult patients. Physicians were selected and invited to participate by the sponsor. Study participants were selected based on their expertise in malnutrition management and their readiness to participate in the study. Moreover, to provide a holistic view of the management of malnutrition in real-world clinical practice in Spain, healthcare professionals from different specialties (digestive, endocrinology and nutrition, geriatrics, hematology, internal medicine, medical oncology, radiation oncology and others) and healthcare areas were invited to participate.

Once the members of the PerceptiONS group had agreed to participate, they were provided with a personal access password by email and the link to the electronic questionnaire posted on a specific website developed for the project. The electronic survey included an electronic informed consent form, which participants had to read and accept before accessing the questionnaire; otherwise, they would not be able to participate in the project. The questionnaire was launched and made available between 1 January 2022 and 31 July 2022.

### 2.2. Electronic Survey

The steering committee developed the content and design of the electronic survey based on a literature review dealing with studies on the management of malnutrition with ONS in patients with a variety of pathologies.

The electronic survey explored each physician’s perception of the experience of five malnourished outpatients that received ONS in their medical practices. The survey questions were grouped into two sections. The first one gathered information regarding the sociodemographic (age, sex and autonomous region) and professional characteristics (specialty and years of experience) of physicians. The second one included three blocks of questions to collect each physician’s perception of the patient’s experience with ONS. The first block contained two questions about the physician’s perception of patient adherence to ONS. The second block comprised five questions about the physician’s perception of patient acceptance or satisfaction. The third included two questions about the physician’s perception of the patient’s clinical improvement after taking the ONS. One of the questions included in this last block was an ad hoc question specific to each ONS. Finally, the basic characteristics of the patient such as age, sex, underlying pathology and time in treatment with the ONS were collected to characterize the sample. In order to obtain a full picture of the management of the malnourished patient in real-world clinical practice, a question was included to determine which pathology justified treatment with ONS (for all patients’ pathologies, see Table S1).

The electronic survey included closed-ended questions (multiple choice) and 4-point Likert-type scale questions (not at all, a little, quite a lot, a lot) (for the full survey, see Table S2 in Supplementary Materials).

Each physician reported their perception of the experience of patients over 18 years of age with a diagnosis of malnutrition; non-palliative situation (>6 months life expectancy); without previous experience of ONS (3 months prior) or home parenteral nutrition; without a high degree of dependency or severe cognitive impairment. Patients started to receive one of the three different ONS at the physician’s discretion: (i) hypercaloric, high-protein peptide-based ONS rich in medium-chain triglycerides (MCT) without fiber; (ii) hypercaloric, high-protein ONS enriched with HMB and fructooligosaccharides (FOS) or (iii) hypercaloric, high-protein diabetes-specific ONS with high mono-unsaturated fatty acids (MUFA), for a minimum of 30 days.

### 2.3. Statistical Analysis

A descriptive analysis using statistical software for data science (STATA V.14.2) was conducted. The mean and standard deviation (SD) were calculated for continuous variables, and frequency (n) and percent (%) were calculated for categorical variables.

The minimum sample size was estimated by using the formula of the population proportion estimation. The criterion of maximum variability was applied, with a 95% confidence interval and a 5% margin of error. A minimum sample size of 385 physicians’ perspectives on patients was required.

Different subgroup analyses were carried out. The first one was according to the ONS prescribed by the physicians, the second one was performed based on the age of the patients (≤65 and >65 years) and the last one according to the underlying pathology (pathologies were grouped into two categories: oncological or non-oncological pathologies).

## 3. Results

A total of 595 physicians were invited to participate in the study, of whom 573 completed the electronic survey and reported their perspectives of 2872 patients. However, due to incomplete survey data or to patients not receiving one of the three study ONS, 349 patients’ experiences were excluded from the statistical analysis. Thus, the final analysis included data reported by 548 physicians on how they perceived the experience of a total of 2516 patients.

Most of the physicians participating were men (63.69%) with a mean age of 39.29 years (SD: 8.59) and a mean experience in the management of malnutrition of 10.36 years (SD: 8.25). Most of the Spanish regions were represented (Andalusia, 27.01%; Madrid, 23.18%; Valencian Community, 12.96%; Castille la Mancha, 6.75%; Castille and Léon, 6.39%, among others). Table 1 summarizes the sociodemographic and clinical characteristics of the physicians participating.

Physicians reported their perception of the experience of 2516 malnourished outpatients (53.58% men; 37.68% older than 75 years) who received the ONS for an average of 85.58 (SD: 24.65) days. Regarding the underlying pathology for which the ONS was prescribed by physicians, the most frequent one was digestive tumor (18.20%), followed by convalescent pluri-pathological patients (17.13%), oncology patients undergoing active chemotherapy or radiotherapy treatment (14.79%) and head and neck tumor/ear–nose–throat (ENT) surgery/maxillofacial (11.72%). Regarding the number of pathologies, 80.6% had one underlying pathology, 14.70% had two and 4.7% had ≥ three pathologies (for all patients’ sociodemographic characteristics, see Table S1).

**Figure 1:**
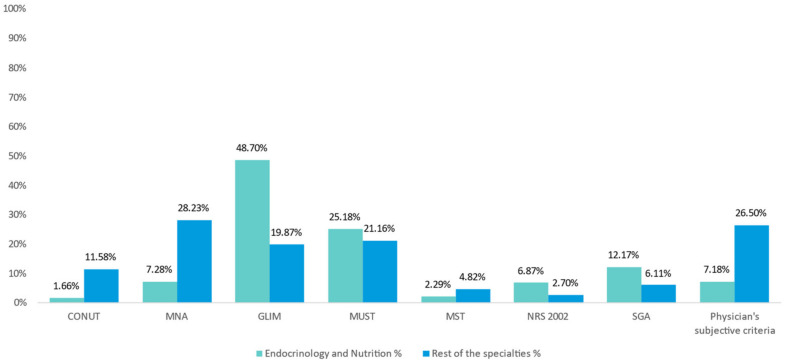
The tool employed to evaluate patient malnutrition by endocrinologists and nutritionists vs. the rest of the specialists (digestive, geriatrics, hematology, internal medicine, medical oncology, radiation oncology and others).

When asked about the most frequent tool employed to evaluate patient malnutrition, the physicians most commonly named GLIM (30.88%), followed by Malnutrition Universal Screening Tool (MUST) (22.69%) and Mini Nutritional Assessment (MNA) (20.23%). A similar pattern of nutritional assessment tool use was observed among endocrinologists and nutritionists (N = 205/548) (48.70% GLIM, 25.18% MUST and 12.17% Subjective Global Assessment, SGA). However, a different pattern was revealed when analyzing the rest of the specialties (N = 343/548) (digestive, geriatrics, hematology, internal medicine, medical oncology, radiation oncology and others). Thus, we found that the most frequently used tool in these specialties was the NMA (28.23%), followed by MUST (21.16%) and GLIM (19.87%). Figure 1 shows the tools employed to evaluate patient malnutrition as well as the differences between endocrinologists and nutritionists versus the rest of the specialties.

### 3.1. Physicians’ Perception of Patient Adherence to ONS

**Figure 2:**
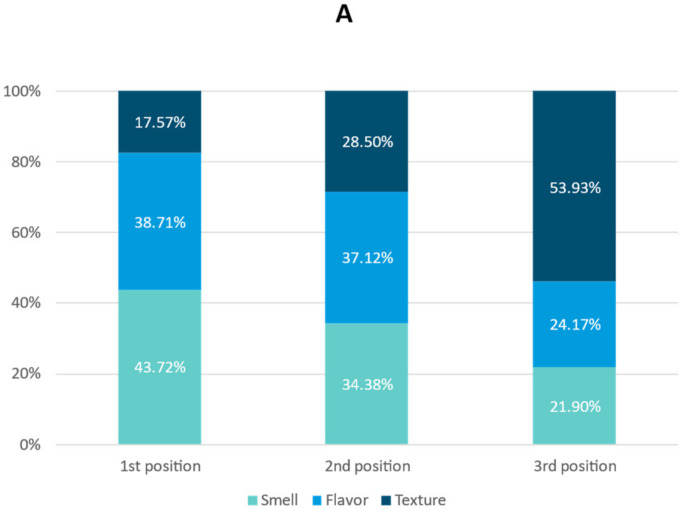

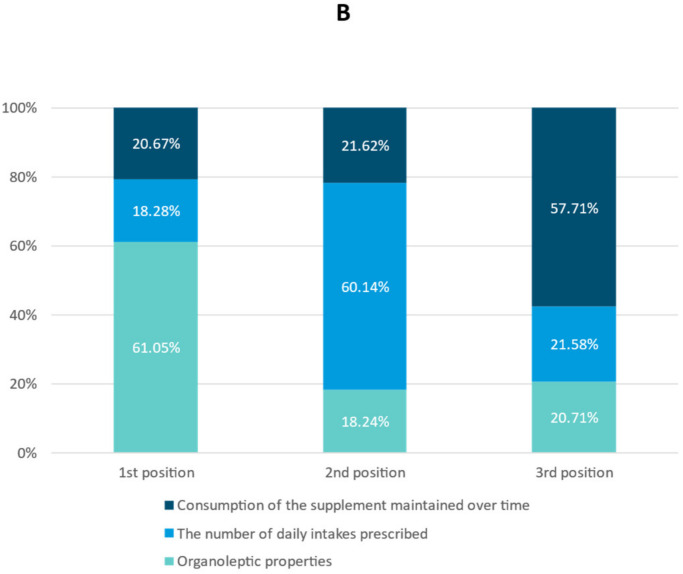
(**A**) Which of these organoleptic properties: smell, flavor or texture, has the most influence on adherence to the nutritional supplement? (**B**) Which of these options: organoleptic properties, the number of daily intakes prescribed or the consumption of the supplement maintained over time, has influenced ONS adherence most positively?

Survey results regarding the physicians’ perception of patient adherence to ONS indicated that 57.11% of patients adhered to over 75% of the prescribed ONS. Smell (43.72%) was the ONS characteristic that most impacted adherence, followed by flavor (37.12%) and texture (53.93%) (Figure 2A). Moreover, physicians considered that the organoleptic properties of ONS exerted the most positive impact on adherence (Figure 2B).

### 3.2. Physicians’ Perception of Patient Acceptance/Satisfaction with ONS

**Figure 3:**
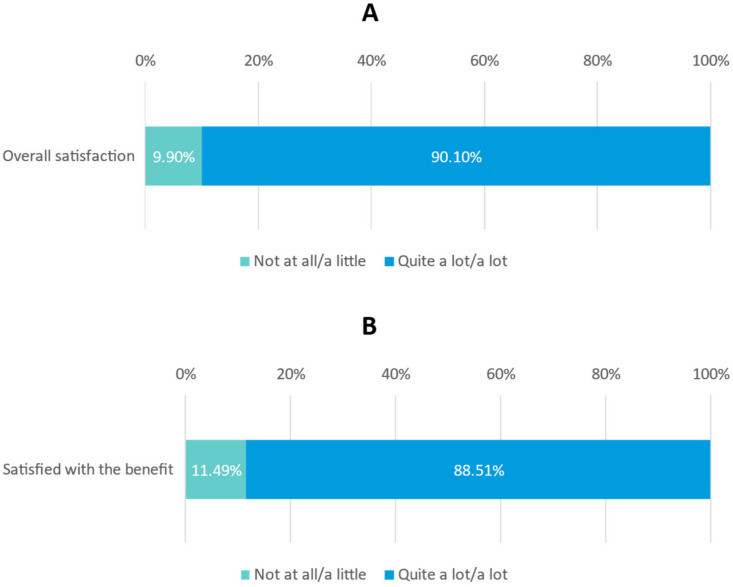

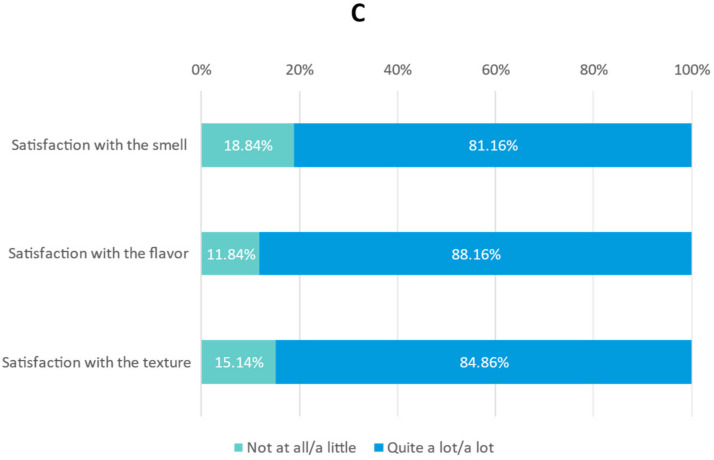
(**A**) Overall patient satisfaction with the ONS received; (**B**) Level of satisfaction with the benefit of the ONS; (**C**) Patient satisfaction with the organoleptic properties.

Most physicians reported that, from their point of view, patient satisfaction with the ONS prescribed was ‘quite a lot’ or ‘a lot’, detailed as follows: overall satisfaction (90.10%) (Figure 3A); benefit of ONS (88.51%) (Figure 3B) and satisfaction with organoleptic properties of the ONS (smell 81.16%, taste 88.16%, texture 84.86%) (Figure 3C).

**Figure 4:**
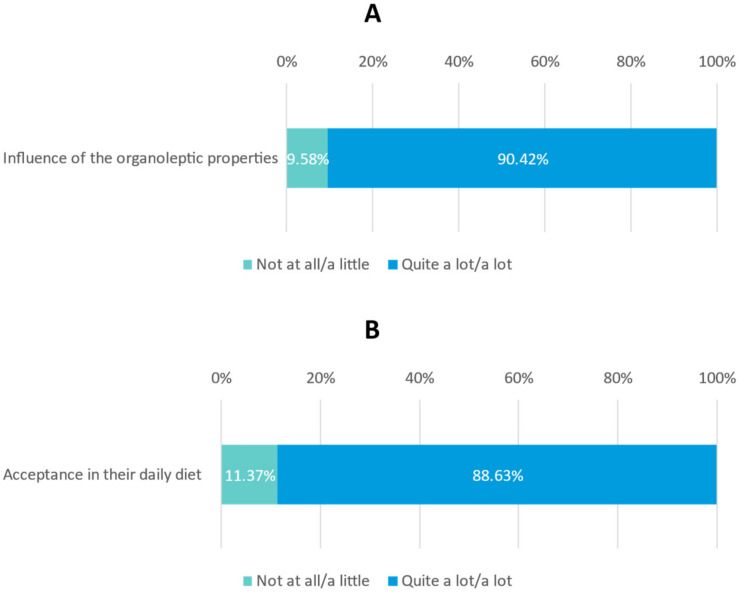
(**A**) Influence of organoleptic properties on patient satisfaction; (**B**) Degree of acceptance of the patient’s ONS in their daily diet.

Moreover, most participants considered that the organoleptic properties influenced patient satisfaction ‘quite a lot’ or ‘a lot’ (90.42%) (Figure 4A) and that patients accepted the ONS in their daily diet ‘quite a lot’ or ‘a lot’ (88.63%) (Figure 4B).

### 3.3. Physicians’ Perception of Patients’ Clinical Improvement after Taking the ONS

**Figure 5:**
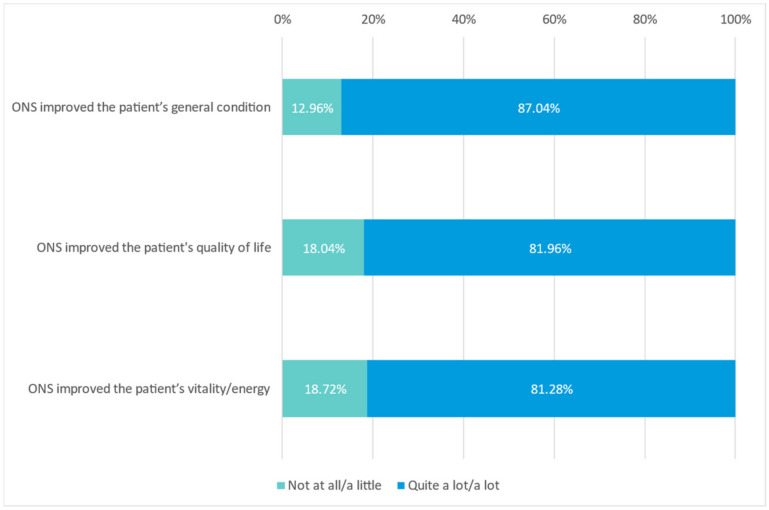
Improvement in the patients’ general condition, vitality/energy and QoL by ONS.

From the physicians’ perception, ONS contributed ‘quite a lot’ or ‘a lot’ to improving the patients’ general condition (87.04%), the patients’ QoL (81.96%) and the patients’ vitality/energy (81.28%) (Figure 5) (for all patients’ clinical improvement, see Table S3).

**Figure 6:**
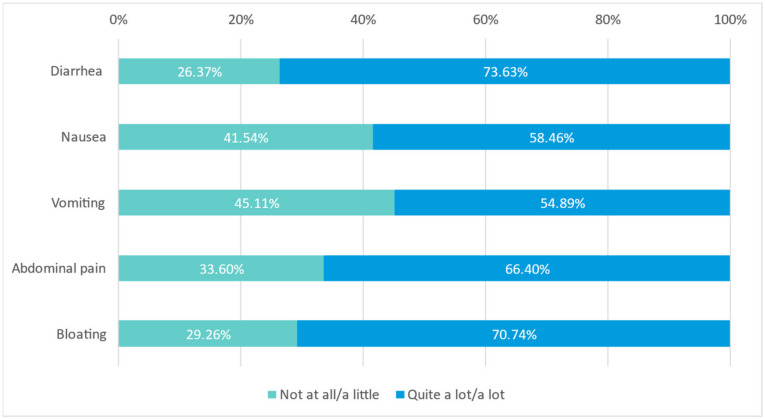
Improvement in the patients’ symptoms (diarrhea, nausea, vomiting, abdominal pain, bloating) by ONS (N = 638 physicians).

A specific question on each ONS was included in the survey. Results for this specific question are shown in Figure 6. Thus, 71.47% of physicians considered that the hypercaloric, high-protein peptide-based ONS rich in MCTs without fiber improved patients’ gastrointestinal discomfort ‘quite a lot’ or ‘a lot’ (71.47%). In addition, physicians considered that for those patients who presented diarrhea (80.25%), nausea (61.13%), vomiting (46.69%), abdominal pain (77.90%) or bloating (78.21%), ONS improved these symptoms ‘quite a lot’ or ‘a lot’: diarrhea (73.63%), nausea (58.46%), vomiting (54.89%), abdominal pain (66.40%) and bloating (70.74%) (Figure 6).

**Figure 7:**
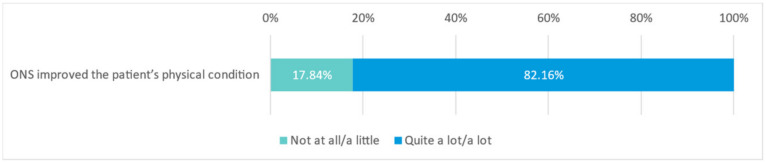
Improvement in the patients’ physical condition by ONS (N = 863 physicians).

Most physicians (82.16%) agreed that the hypercaloric, high-protein ONS enriched with HMB and FOS improved the patients’ physical condition ‘quite a lot’ or ‘a lot’ (Figure 7).

**Figure 8:**
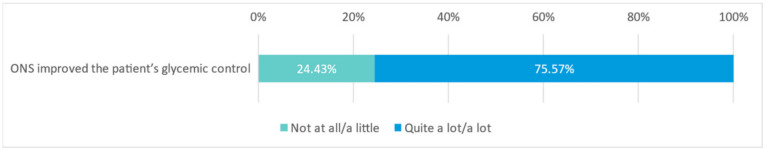
Improvement in the patients’ glycemic control by ONS (N = 749 physicians).

Finally, 75.57% of participants indicated that the diabetes-specific hypercaloric, high-protein ONS with high MUFA contributed to better glycemic control ‘quite a lot’ or ‘a lot’ (Figure 8).

### 3.4. Physician Satisfaction

After their experience prescribing ONS, almost all the physicians stated they would prescribe the same ONS again, in 96.4% of the cases.

### 3.5. Subgroups Analyses

No differences were observed according to the type of ONS [(i) hypercaloric, high-protein peptide-based ONS rich in MCT without fiber; (ii) hypercaloric, high-protein ONS enriched with HMB and FOS and (iii) hypercaloric, high-protein diabetes-specific ONS with high MUFA].

The subgroup analysis according to patients’ age (≤65 and >65 years) assessed differences regarding the following questions: how much has the ONS contributed to (i) improving the patient’s autonomy/functionality, (ii) achieving weight gain in the patient and (iii) the patient’s degree of adherence to the prescribed treatment? Results showed no differences between groups.

The last subgroup analysis carried out based on patients’ underlying pathology (oncology or non-oncology patients) evaluated the responses to the following questions: (i) how much has the ONS contributed to influencing the patient’s daily intake, (ii) the patient’s satisfaction with the taste of the ONS, (iii) the patient’s degree of acceptance of ONS in their daily diet and (iv) the degree of adherence to the prescribed treatment? Results did not detect any differences (for all subgroup analysis results, see Table S4).

## 4. Discussion

PerceptiONS is a study describing the perception of physicians with experience in the management of ONS in malnourished patients (mean 10.36 years; SD: 8.25) in Spain. This electronic survey provided representative data on the physicians’ opinions.

Nearly 38% of participants were specialists in endocrinology and nutrition (37.41%), who routinely used validated screening methods (GLIM, MUST or MNA, a total of 73.81%) to detect patients who would benefit from an ONS-based intervention. Only 7.2% of endocrinologists and nutritionists did not employ some screening tools. This contrasts with rest of specialists, who did not use a validated screening malnutrition tool in 26.5% of cases. Barriers to the use of appropriate screening methods may be the lack of resources (time, staff, etc.), lack of training or the lack of care pathways to guide action after screening; however, this was not evaluated [25].

Participants reported their perceptions regarding the experience of 2516 patients receiving ONS for almost three months (90% for a duration of 60–120 days). Most patients suffered from digestive neoplasms or a multi-pathological condition, thus broadly representing real-world clinical practice. In addition, 63.47% of patients were over 66 years old, with the main part of the sample >75 years (37.68%). A subset of elderly patients probably provides more evidence for using ONS in fragile subjects, in that older age implies greater risk of fragility.

Our data showed that the participating physicians perceived that most patients (57.11%) adhered correctly to ONS usage, consuming at least 75% of the prescribed dose. According to the physicians, smell and flavor were the two main factors conditioning adherence. Organoleptic characteristics were the main features favoring adherence, in the physicians’ opinion. Adherence to ONS reported in our study based on the physicians’ perception was higher than previously described rates. In a systematic review and meta-analysis performed in 2019 by Gea-Cabrera et al. [23], high adherence was only found in hospitalized patients, where it reached 80%, whereas adherence rates in outpatients tended to be around 50%. This variance could be explained by methodological and study population differences between the two studies, as the aforementioned meta-analysis included studies in which the main pathologies considered were represented less frequently in our study, such as type 2 diabetes, Chron’s disease and obesity, and an exclusively geriatric population. Additionally, such variance could relate to improvements in the organoleptic characteristics due to new developments, which may have contributed to enhancing adherence rates.

In general, our results suggest that physicians considered that patients were satisfied (90.10%) with the ONS, deeming that organoleptic properties influenced patient satisfaction ‘quite a lot’ or ‘a lot’ (90.40%). A high satisfaction rate with ONS was expected, given that previous studies based on less convenient enteral feeding strategies reported they had been well tolerated by patients. A prospective study including 149 patients (80% male) with gastrointestinal neoplasms (76% adenocarcinomas) treated via jejunostomy reported a 96% compliance rate and a mean satisfaction score >70 ± 11/100 (QLQ-C30 questionnaire) [26].

Physicians’ appreciation of the effect exerted by ONS on clinical improvement was remarkably positive on all the parameters studied, including patients’ general condition, QoL and vitality. The results of our study show the positive effects of ONS on improving patients’ functional status, thereby dispelling the existing doubts on whether clinical trial findings translate to real-world clinical practice settings [27]. Regarding gastrointestinal symptoms management, physicians considered that the peptide-based ONS contributed to reducing diarrhea, abdominal pain and bloating. A recent observational study performed in 19 medical sites in Spain reported similar results in 90 malnourished adult patients with gastrointestinal malabsorption symptoms [28]. It showed that peptide-based ONS reduced symptoms and improved patients’ nutritional status [28]. The ONS enriched with HMB and FOS were perceived as effective for recovering the patient’s physical condition. Similarly, the diabetes-specific ONS were deemed suitable for achieving glycemic control.

Tolerability is a challenge to maintaining enteral nutrition feeding. Thus, in an observational, multicenter study conducted in Spain involving a total of 148 patients and 114 physicians, tolerability was paramount for patients receiving home enteral nutrition (HEN). Significantly, younger patients had stronger preferences for tolerability, whereas elderly patients (≥75 years) were more concerned about the ease of handling. By comparison, physicians assigned a greater relative importance to tolerability, nutrition and calories compared to patients (*p*  =  0.002) [29]. An observational, prospective, multicenter study (n = 90) conducted in Spain evaluated nutritional status and compliance with a high-protein peptide-based ONS in a malnourished population with gastrointestinal intolerance over a 12-week period [30]. Gastrointestinal tolerance was good (75.0% of patients did not have abdominal pain or pain improved), with a decrease in the number of episodes of diarrhea, abdominal distension, nausea and vomiting. Stool consistency also improved during treatment. The adherence rates were in line with the tolerance rate (75.5%) [29].

Consistent with the high adherence rates, good clinical benefits and tolerability perceived, almost all physicians (96.40%) stated they would use the same ONS in similar patient profiles in the future. Our results provide evidence in favor of prescribing ONS to malnourished patients, supporting their use as a strategy to combat malnutrition.

One of the main strengths of the PerceptiONS study lies in its large study sample. Our results describe the perception of 548 physicians regarding the experience of 2516 malnourished outpatients receiving ONS in the Spanish public healthcare system. In addition, our results show that there are no differences in the use of the different nutritional supplements according to either the patient’s age or underlying pathology. This suggests that our results could be extrapolated to other patients. However, this study has some limitations, some of which are inherent to the study design. Firstly, all the parameters studied were assessed from each physician’s subjective perception, which may potentially have led physicians to misinterpret their patients’ experiences. Although the physician’s perception may not fully reflect the patient’s perception, the information provided will be useful to better understand and optimize patient management in this context.

Secondly, the study is based on an ad hoc questionnaire, making it more difficult to compare these results with similar investigations. Thirdly, the study was carried out in Spain; therefore, our findings may not be extrapolated to other settings. Finally, it would also have been desirable to perform diagnostic tests to quantify certain clinical features. For instance, ultrasound or bioimpedanciometry could be used to measure muscular mass, and this information could be compared with the improvements perceived by the physicians in the patient’s condition and strength. Further studies exploring the patients’ perception of the issues addressed in this project would be of great interest.

## 5. Conclusions

To our knowledge, PerceptiONS is the first study exploring physicians’ perceptions of malnourished outpatients prescribed ONS regarding adherence, acceptance/satisfaction, tolerability, and benefits. From the physicians’ perspective, most patients adhered correctly to the ONS prescribed. Smell and flavor were deemed the main factors conditioning ONS adherence. In addition, physicians perceived that ONS were well tolerated, mainly due to their organoleptic characteristics, and considered that they improved patients’ general condition, energy and QoL.

## Figures and Tables

**Table 1:** Physicians’ sociodemographic and clinical characteristics (n = 548).

Characteristics	% or Mean (SD)
Age, years, mean (SD)	39.39 (8.59)
Male, %	63.69
Experience, years, mean (SD)	10.36 (8.25)
Medical Specialty, %	
Digestive	7.30
Endocrinology and Nutrition	37.41
Geriatrics	14.23
Hematology	0.91
Internal Medicine	16.97
Medical Oncology	5.66
Radiation Oncology	10.77
Other *	6.75

* Other (>2%): General and digestive surgery and family and community medicine.

## Data Availability

The data presented in this study are available on request from the corresponding author.

## Supplementary Materials

The following supporting information can be downloaded at: https://www.mdpi.com/article/10.3390/nu15051219/s1, Table S1: Patients’ sociodemographic characteristics; Table S2: Complete questionnaire; Table S3: Physicians’ perspective on patient’s clinical improvement after taking the ONS; Table S4: Subgroup analyses.

## Appendix A

PerceptiONS group members: Elena Atienza Sánchez (Hosp. Torrejón), Jimena Abiles Osinaga (Hosp. Costa Del Sol), Jose Abuin Fernández (Complejo Hosp. Regional Universitario Málaga), Miguel Aganzo Yeves (Hosp. Universitario Fundación Jimenez Diaz), Ana Lucía Agudo Beato (Complejo Hosp. Virgen Macarena), Fernando Aguilar Rodriguez (Complejo Hosp. Universitario 12 De Octubre), María Del Mar Aguilar Martinez (Hosp. De Poniente), Valeria Alcántara Aragón (Complejo Hosp. Universitario Marqués De Valdecilla), Rubén Alcantud Córcoles (Complejo Hosp. Universitario Albacete), María Alcantud Ibáñez (Hosp. Universitario Infanta Leonor), Jaime Alcober Perez (Hosp. General Universitari D’Alacant), Irene Alda Bravo (Complejo Hosp. Universitario 12 De Octubre), Mirian Alejo Ramos (Hosp. Del Bierzo), María Asunción Algarra García (Hosp. De La Marina Baixa), Ana Almendro Nogueres (Hosp. Marina Salud), Fernando Alonso Ecenarro (Consorcio Hosp. General Universitario Valencia), Mario Zacarias Alonso Vilasuso (Complejo Hosp. Universitario Canarias), Ana Isabel Alonso García (Complejo Hosp. Universitario Asturias), Javier Alonso Ramirez (Hosp. Doctor Jose Molina Orosa), Macarena Alpañés Buesa (Hosp. Universitario Ramon Y Cajal), María Chian Alvarez Martin (Complejo Hosp. Cartagena), Carlos Manuel Alzas Teomiro (Complejo Hosp. Reina Sofia), Enrique Amaya Escobar (Hosp. Universitario Rey Juan Carlos), Angela Amengual Galbarte (Hosp. Universitario Rey Juan Carlos), María Del Carmen Andreo López (Hosp. Universitario San Cecilio), María Andreo Galera (Hosp. Universitario Torrevieja), Joaquín Antón Martínez (Complejo Hosp. Cáceres), Roberto José Añez Ramos (Complejo Hosp. Gregorio Marañón), Carmen Aragón Varela (Hosp. Universitario Fundación Jimenez Diaz), Juan José Arechederra Calderón (Hosp. Universitario Guadalajara), María Caridad Arenas Martinez (Hosp. Universitario Puerta De Hierro Majadahonda), María Argente Pla (Complejo Hosp. La Fe), Estefanía Arias Muñana (Hosp. Universitario Infanta Sofia), Jose Antonio Ariza Jimenez (Complejo Hosp. Nuestra Señora De Valme), María Elena Arjonilla Sampedro (Hosp. General Universitario Morales Meseguer), Claudia Arnas Leon (Complejo Hosp. Universitario Gran Canaria Doctor Negrín), Elena Arregui Lopez (Hosp. General Universitario De Ciudad Real), Miren Arteaga Mazuelas (Hosp. García Orcoyen), Rosa Ana Ashbaugh Enguidanos (Hosp. Universitario Principe De Asturias), Verónica Ávila Rubio (Hosp. Universitario San Cecilio), Luisa Ayala Corao (Fund. Hosp. Son Llatzer), Javier Azaña Gomez (Hosp. Universitario Clínico San Carlos), Susana Bacete Cebrian (Hosp. Universitario Infanta Leonor), Marina Baez Rivas (Hosp. Universitario Puerto Real), Juan De Dios Barranco Ochoa (Complejo Hosp. Jaén), Pilar Barrio Dorado (Hosp. Universitario Fundación Jimenez Diaz), Elvira Barrio Escribano (Hosp. Universitario Del Henares), Laura Bartolomé Hernández (Hosp. Universitario Fundación Jimenez Diaz), Natalia Bassy Iza (Hosp. Universitario Guadalajara), Cristina Bautista Galán (Hosp. Antequera), Glenda María Bautista Suarez (Complejo Hosp. Universitario Gran Canaria Doctor Negrín), Inmaculada Beato Tortajada (Hosp. General Universitario De Castellón), Gessy Bellerive. (Hosp. General La Mancha Centro), Estela Benito Martinez (Hosp. Comarcal De Urduliz-Alfredo Espinosa), Miguel Angel Berenguer Frances (Complejo Hosp. La Fe), Laura Bermejo García (Hosp. Universitario Clínico San Carlos), María Berrio Miranda (Hosp. Universitario San Cecilio), Ariadna Besga Basterra (Complejo Hosp. Universitario Araba), Raquel Besse Díaz (Hosp. Universitario Ramon Y Cajal), Manuel Luis Blanco Villar (Complejo Hosp. Torrecardenas), Benjamín Blanco Ramos (Hosp. General Universitario De Elda Virgen De La Salud), Carmen Botella Prieto (Hosp. General Universitari D’Alacant), Irene Bretón Lesmes (Complejo Hosp. Gregorio Marañón), Francisco Brun Romero (Hosp. Universitario Puerta Del Mar), Almudena Cabero Martínez (Complejo Hosp. Salamanca), Luis Cabeza Osorio (Hosp. Universitario Del Henares), Lara Cacace. (Hosp. Costa Del Sol), Aida Cadenas Gonzalez (Hosp. Galdakao-Usansolo), Alexandra Cadova Cadova (Hosp. Cabueñes), Eugenia Caffarena Funcia (Complejo Hosp. Especialidades Virgen De La Victoria), Alfonso Calañas Continente (Complejo Hosp. Reina Sofia), Ismael Calero Paniagua (Hosp. Virgen De La Luz), Carlos Callen Roche (Hosp. Virgen De La Luz), Julio Calvete Cárdenas (Hosp. Universitario Puerta Del Mar), Fernando Calvo Gracia (Hosp. Clínico Universitario Lozano Blesa De Zaragoza), Salvador Calzado Baeza (Hosp. General Básico Santa Ana), Raquel Camargo Camero (Complejo Hosp. Especialidades Virgen De La Victoria), Miguel Camblor Álvarez (Complejo Hosp. Gregorio Marañón), Juana María Cano Cano (Hosp. General Universitario De Ciudad Real), José Canto Mangana (Hosp. De Poniente), Verónica Cañón García (Complejo Hosp. Universitario Marques De Valdecilla), Amaya Capón Sáez (Complejo Hosp. Navarra), Juan Jesús Carabantes Rueda (Hosp. Antequera), Blanca Carballido De Miguel (Hosp. Universitario Puerta De Hierro Majadahonda), Inmaculada Carmona Álvarez (Complejo Hosp. Gregorio Marañón), Carlos Carrascal Gordillo (Complejo Hosp. Universitario Asturias), Pablo Carrasco Lara (Hosp. Comarcal El Escorial), Pamela Carrillo García (Complejo Hosp. Universitario La Paz), Guilherme Carvalho Monteiro (Complejo Hosp. Salamanca), Margarita Casado Jimenez (Hosp. Universitario De La Princesa), Luis Casamayor Escriva (Hosp. Ernest Lluch Martin), Cristina Casares Merino (Complejo Hosp. Cáceres), Carmen Castellanos Lluch (Complejo Hosp. La Fe), Sandra Castellanos Viñas (Complejo Hosp. León), Máximo Castilla Selva (Complejo Hosp. Universitario Canarias), Trinidad Castillo García (Hosp. General Universitari D’Alacant), Iciar Castro De La Vega (Hosp. General Universitario De Castellón), Manuel Cayón Blanco (Hosp. De Jerez), Abel Cedeño Veloz (Complejo Hosp. Navarra), Laura Cerezo Padellano (Hosp. Universitario De La Princesa), Rafael Cerezo Vidal (Hosp. Universitario Vinalopó), Rocío Chamorro Mohedas (Hosp. Universitario Puerta Del Mar), Jose Luis Chicon Paez (Complejo Hosp. Llerena-Zafra), María Jesus Chinchetru Ranedo (Complejo Hosp. San Pedro La Rioja), María Cienfuegos-Jovellanos Romero (Hosp. Cabueñes), Soralla Civantos Modino (Hosp. Universitario Fuenlabrada), Migle Civera Andrés (Hosp. Clínico Universitario Valencia), Carlos Colato López (Hosp. Universitario Guadalajara), Sonia Colomina Monzo (Hosp. General Universitari D’Alacant), Cristina Comi Diaz (Complejo Hosp. Universitario Gran Canaria Doctor Negrín), Teresa Concepcion Medina (Complejo Hosp. Nuestra Señora Candelaria-Ofra), Cristina Conejos Bono (Hosp. Universitario Doctor Peset), Cristina Contreras Pascual (Complejo Hosp. Miguel Servet), Isabel María Cornejo Pareja (Complejo Hosp. Especialidades Virgen De La Victoria), Luz Cornejo Olivas (Hosp. Marina Salud), Uriel Corro Verde (Complejo Hosp. Universitario Marqués De Valdecilla), Alba María Costa Grille (Hosp. Universitario Getafe), Cristina Cruces Vega (Hosp. Infanta Elena), Jose Alfonso Cruz Conde (Hosp. Universitario De La Princesa), Alejandro Cruz Carvajal (Complejo Hosp. Universitario Asturias), Cristina Cruz Muñoz (Complejo Asist. Campo De Gibraltar), Laura Cuadrado Clemente (Hosp. Clínico Universitario Valladolid), Sara Custodio Cabello (Hosp. Torrejón), Carolina Dassen De Monzo (Hosp. Universitario Fundación Jimenez Diaz), Betty Luz Claudia Davies Urizar (Hosp. Universitario Getafe), Adela Dávila Jerez (Complejo Hosp. Nuestra Señora Candelaria-Ofra), Luis Pablo De Benito Cordón (Hosp. Virgen De La Luz), Víctor De Diego Sola (Complejo Hosp. Universitario Araba), Miguel Ángel De Jorge Turrion (Hosp. Cabueñes), María De La Puente Martin (Hosp. Universitario Severo Ochoa), Beatriz De Leon Fuentes (Hosp. Universitario Cruces), Daniel De Luis Román (Hosp. Clínico Universitario Valladolid), Beatriz De Tapia Majado (Complejo Hosp. Salamanca), Florentino Del Val Zaballos (Hosp. General La Mancha Centro), Juan Delgado De La Cuesta (Complejo Hosp. Virgen Del Rocío), Esther Delgado García (Hosp. Clínico Universitario Valladolid), Luz Delgado Dominguez (Complejo Hosp. Nuestra Señora Candelaria-Ofra), Nuria Delgado Jimenez (Hosp. Universitario Basurto), Ana Delgado Maroto (Complejo Hosp. Torrecardenas), Laura Díaz Gómez (Hosp. De Jerez), Alberto Díaz Jimenez (Complejo Asist. Campo De Gibraltar), Claudia Marcela Díaz Silvera (Hosp. Universitario Fundación Jimenez Diaz), Macarena Diaz De Bustamante De Ussia (Hosp. Universitario Puerta De Hierro Majadahonda), Soledad Dominguez Mendoza (Complejo Hosp. Navarra), Viyey Kishore Doulatram Gamgaram (Complejo Hosp. Regional Universitario Málaga), Maddalen Dublang Irazabal (Hosp. Galdakao-Usansolo), Iris El Attar Acedo (Complejo Hosp. Torrecardenas), Javier Ena Muñoz (Hosp. De La Marina Baixa), Cristina Escorial Moya (Complejo Hosp. Virgen Del Rocío), Irene Esparcia Arnedo (Complejo Asist. Universitario Burgos), Ricardo Espinosa Calleja (Hosp. San Juan De Dios Del Aljarafe), Jose Luis Esquinas Requena (Hosp. General Universitario De Ciudad Real), Roció Estepa Cabello (Hosp. De Jerez), María Ana Estornell Gualde (Complejo Hosp. La Fe), Sara Estrada Dorronsoro (Hosp. Galdakao-Usansolo), Lourdes Rosario Evangelista Cabrera (Hosp. Universitario Severo Ochoa), María José Fabia Valls (Hosp. Universitario Doctor Peset), Sandra Falagan Martinez (Hosp. Universitario Infanta Sofia), Ramon Feria Bataller (Hosp. Virgen De La Luz), Sara Fernández Villaseca (Complejo Hosp. Universitario 12 De Octubre), Beatriz Fernández Medina (Complejo Hosp. Especialidades Virgen De La Victoria), Alejandra Fernández Pordomingo (Complejo Hosp. Salamanca), Jose David Fernández Arias (Hosp. Comarcal De Melilla), María José Fernández Cordero (Hosp. General Juan Ramon Jimenez), Lucia Fernández Arana (Hosp. Universitario Puerta De Hierro Majadahonda), Arancha Fernández Orgiler (Hosp. De La Marina Baixa), Virginia Esperanza Fernández Ruiz (Hosp. Clínico Universitario Virgen De La Arrixaca), Joaquín Andrés Fernández Muñoz (Hosp. La Inmaculada), Marta Fernández Álvarez (Hosp. La Inmaculada), Raquel Mercedes Fernández Garzón (Complejo Hosp. Torrecardenas), Carmen Fernández Lopez (Hosp. Universitario Cruces), Jose María Fernández Recio (Complejo Hosp. Universitario Badajoz), Yvonne Fernández Cagigao (Hosp. Universitario Fundación Jimenez Diaz), Encarnación Fernández Camacho (Hosp. General Universitario De Castellón), María Luisa Fernández Soto (Hosp. Universitario San Cecilio), Javier Fernández Rivera (Complejo Hosp. Virgen Del Rocío), Javier Fernández-Cuervo Lorente (Hosp. General Universitario De Castellón), María Luisa Ferrandez Millan (Hosp. Clínico Universitario Lozano Blesa De Zaragoza), Laura Ferrera Alayón (Complejo Hosp. Universitario Gran Canaria Doctor Negrín), Alberto Ferreras García (Complejo Asist. Zamora), Paula Ferrero León (Hosp. San Juan De Dios Del Aljarafe), Eugeni Figols Ibañez (Hosp. La Plana), Luna Florencio Ojeda (Hosp. General Juan Ramon Jimenez), Henedina Flores Moreno (Hosp. Comarcal De La Axarquia), Pablo Flors Villa Verde (Hosp. Lluis Alcanyis), Rosa María Folgado De La Fuente (Hosp. Universitario La Ribera), Alicia Frances Muñoz (Hosp. General Universitario De Castellón), Fatima A. Francisco Sano (Complejo Hosp. Salamanca), José María Frutos Perez (Hosp. Francesc De Borja), Sergio Tomas Fuentes Tudanca (Hosp. Universitario Rey Juan Carlos), Miriam Gabella Martin (Hosp. Universitario Rio Hortega), Francisco Gallardo Sanchez (Hosp. De Poniente), Celia Gallego Méndez (Hosp. Clínico Universitario Valencia), Fabiola Gallego Gamero (Complejo Hosp. Llerena-Zafra), Katherine García Malpartida (Complejo Hosp. La Fe), María Elena García Morales (Complejo Hosp. Nuestra Señora Candelaria-Ofra), Ignacio García Puente (Complejo Hosp. Universitario Gran Canaria Doctor Negrín), Camila García Volpe (Hosp. De Sant Joan Despi Moises Broggi), Blanca García García (Hosp. General San Jorge), María Rosario García Martin (Hosp. Universitario Severo Ochoa), María Paola García Coves (Hosp. Comarcal De La Vega Baja), Elena García Ruiz (Complejo Asist. Campo De Gibraltar), Laura García Pereña (Hosp. Universitario San Cecilio), Rosa María García Moreno (Complejo Hosp. Universitario La Paz), Claudia García Lobato (Complejo Hosp. Universitario Badajoz), María Del Carmen García Marín (Hosp. La Inmaculada), Lourdes García Blasco (Complejo Hosp. Universitario Albacete), Alejandro García Martínez (Hosp. De Jerez), María García Duque (Hosp. Universitario Rio Hortega), Rocío García Serrano (Hosp. San Juan De Dios Del Aljarafe), Yaiza García Delgado (Complejo Hosp. Universitario Insular Materno Infantil), Víctor Garcia-Hierro Gonzalez Regueral (Complejo Asist. Universitario Burgos), Noelia Garcia-Talavera Spin (Hosp. General Universitario Morales Meseguer), Blanca Garmendia Prieto (Complejo Hosp. Universitario La Paz), Fernando Garrachón Vallo (Complejo Hosp. Virgen Macarena), Pilae Geraldo Perez (Hosp. Lluis Alcanyis), Patrícia Gili Martinez-Meco (Hosp. Virgen De La Luz), Mariana Gomes Porras (Complejo Hosp. Regional Universitario Málaga), Mercedes Gomez Hernández (Hosp. San Juan De Dios Del Aljarafe), Roberto Gomez Diaz (Hosp. General Universitario De Ciudad Real), Emilia Gómez Hoyos (Hosp. Clínico Universitario Valladolid), Inés Gómez Molins (Hosp. Universitario Fundación Alcorcón), Laura Gomez-Escolar Viejo (Hosp. De La Marina Baixa), Beatriz Gonzalez Aguilera (Complejo Hosp. Virgen Macarena), Mikel Gonzalez Fernández (Hosp. Clínico Universitario Lozano Blesa De Zaragoza), Paloma Gonzalez Lázaro (Hosp. General La Mancha Centro), Elena Gonzalez García (Complejo Hosp. Universitario La Paz), Víctor Gonzalez Sanchez (Hosp. Universitario Fundación Alcorcón), Javier González García (Hosp. La Inmaculada), Ana María González Ageitos (Complejo Hosp. Toledo), Alba González Castro (Complejo Asist. Universitario Burgos), Aranzazu González Vicente (Hosp. Universitario Torrevieja), Irene González Niño (Hosp. Universitario Ramon Y Cajal), Montserrat Gonzalo Marín (Complejo Hosp. Regional Universitario Málaga), Irene Gonzalo Montesinos (Hosp. Universitario Fuenlabrada), María Gonzalo Lazaro (Complejo Hosp. Navarra), Beatriz Grandal Leirós (Complejo Hosp. Universitario Canarias), Concepción Grau Jiménez (Complejo Hosp. Toledo), Mora Guardamagna Guisasola (Complejo Hosp. Especialidades Virgen De La Victoria), Juan Manuel Guardia Baena (Hosp. Virgen De Las Nieves General), Elena Guerra Del Rio Cardenes (Complejo Hosp. Universitario Gran Canaria Doctor Negrín), Laura Guerrero Casanova (Complejo Hosp. Universitario Gran Canaria Doctor Negrín), Tania Guevara Guevara (Hosp. Universitario Getafe), Eddy Gutierrez Damia (Hosp. Francesc De Borja), Lucia Gutierrez Bayard (Hosp. Universitario Puerta Del Mar), Laura Guzman Gomez (Hosp. Universitario Fundación Jimenez Diaz), Carlos Guzman Carmona (Complejo Hosp. Don Benito-Villanueva De La Serena), Samia Hallouch Toutouh (Complejo Hosp. Torrecardenas), Ana Hernández Machancoses (Consorcio Hosp. General Universitario Valencia), Ana Hernández Moreno (Complejo Hosp. Navarra), Pablo Hernández Zegarra (Hosp. General La Mancha Centro), José Miguel Hernández Rey (Complejo Asist. Campo De Gibraltar), Alvaro Hernández Martínez (Complejo Hosp. Torrecardenas), Álvaro Hernández Pérez (Complejo Asist. Ávila), Sandra Herranz Antolín (Hosp. Universitario Guadalajara), Aura Dulcinea Herrera Martínez (Complejo Hosp. Reina Sofia), Jorge Eduardo Herrera Parra (Complejo Hosp. Universitario Asturias), Elena Hervas Abad (Complejo Hosp. Cartagena), Ana Horcajada Ruiz (Hosp. Universitario De La Princesa), Eduardo Hortelano Pardo (Complejo Hosp. Universitario Araba), José María Huguet Malaves (Consorcio Hosp. General Universitario Valencia), Flavia Lorena Hünicken Torrez (Complejo Asist. Ávila), Luis Ibáñez Muñoz (Complejo Hosp. Virgen Macarena), Miriam Ibars Morales (Complejo Hosp. Torrecardenas), Jose Antonio Irles Rocamora (Complejo Hosp. Nuestra Señora De Valme), Olatz Izaola Jauregui (Hosp. Clínico Universitario Valladolid), María Elena Jerez Arzola (Hosp. Doctor Jose Molina Orosa), Ana Jimenez Portilla (Consorcio Hosp. General Universitario Valencia), Laura Jimenez De La Cruz (Hosp. Virgen De La Luz), Sara Jiménez Blanco (Hosp. Universitario De La Princesa), Clara Joaquín Ortiz (Hosp. Universitario Germans Trias I Pujol), Esther Jorda Sorolla (Hosp. Clínico Universitario Valencia), Lucia Jorge Huerta (Complejo Hosp. Universitario 12 De Octubre), Sonia Junquera Bañares (Complejo Hosp. Universitario La Paz), Alicia Justel Enriquez (Hosp. Universitario De La Princesa), Gorgios Kiryakos (Complejo Hosp. Cartagena), Carolina Knott Torcal (Hosp. Universitario De La Princesa), María Lainez Lopez (Hosp. General Juan Ramon Jimenez), Julio Jose Lambea Sorrosal (Hosp. Clínico Universitario Lozano Blesa De Zaragoza), Soraya Lanes Iglesias (Complejo Hosp. San Pedro La Rioja), Carmen María Lara Rojas (Hosp. De Poniente), Beatriz Lardiés Lardiés (Hosp. General Obispo Polanco), Laura Larran Escandon (Hosp. Universitario Puerta Del Mar), Jose Lazaro Pérez Calle (Hosp. Universitario Fundación Alcorcón), Marta Lázaro Sáez (Complejo Hosp. Torrecardenas), Antonio Lazo Prados (Hosp. Universitario San Cecilio), Jesús Leal Téllez (Complejo Asist. Campo De Gibraltar), Soraya Leon Idougourram (Complejo Hosp. Reina Sofia), Ana Lerma Verdejo (Hosp. General Nuestra Señora Del Prado), Silvia Llopis Salinero (Hosp. Universitario Infanta Leonor), Alfonso Lluna Carrascosa (Hosp. Universitario San Cecilio), Alberto Lopez García (Hosp. Universitario Fundación Jimenez Diaz), María Josefa Lopez Lopez (Hosp. General Universitario Morales Meseguer), Jose Antonio Lopez Medina (Complejo Hosp. Especialidades Virgen De La Victoria), Alfonso Lopez Alba (Hosp. Cabueñes), María Eugenia López Valverde (Hosp. General Juan Ramon Jimenez), Daniel López Wolf (Hosp. Universitario Fundación Alcorcón), Juan José López Gómez (Hosp. Clínico Universitario Valladolid), Luis López Penabad (Hosp. Universitario Sant Joan D’Alacant), José López Sendón (Hosp. Universitario Ramon Y Cajal), Fran López Rodriguez-Arias (Hosp. General Universitario Elche), Yaiza López Plasencia (Complejo Hosp. Universitario Insular Materno Infantil), Gonzalo Lopez- Medel Marina (Hosp. Universitario Clínico San Carlos), Concepcion Losada Morell (Hosp. Infanta Margarita), Rubén Lovatti Gonzalez (Hosp. Universitario Puerta De Hierro Majadahonda), Victoria Luna Lopez (Hosp. Virgen De Las Nieves General), Alejandro Macein Rodriguez (Hosp. Universitario Clínico San Carlos), Inmaculada Maestu Maiques (Hosp. Universitario Doctor Peset), María Del Carmen Mafe Nogueroles (Hosp. Marina Salud), María Maiz Jimenez (Complejo Hosp. Universitario 12 De Octubre), Cristina Maldonado Ubeda (Hosp. De Poniente), María Elena Mansilla Rodríguez (Hosp. Infanta Elena Huelva), Gregorio Manzano García (Complejo Hosp. Reina Sofia), Ana Belén Mañas Martínez (Hosp. Ernest Lluch Martin), Clara Marcuello Foncillas (Hosp. Universitario Clínico San Carlos), Amelia Mari Sanchis (Complejo Hosp. Navarra), Juanjose Marín Peñalver (Hosp. De La Vega Lorenzo Guirao), Luis Marín Martinez (Complejo Hosp. Cartagena), Jorge Marrero Frances (Hosp. Universitario Fuenlabrada), Juan Luis Marti Ciriquian (Hosp. General Universitari D’Alacant), Elvira Martin De La Torre (Complejo Asist. Universitario Burgos), Elisa Martin De Francisco (Hosp. Comarcal El Escorial), María Del Mar Martin Rodriguez (Hosp. Virgen De Las Nieves General), Alba Martín Gonzalez (Complejo Hosp. Universitario 12 De Octubre), Margarita Martín Martín (Hosp. Universitario Ramon Y Cajal), Manuel Martín López (Hosp. General Juan Ramon Jimenez), Rubén Ángel Martín Sánchez (Hosp. Universitario Clínico San Carlos), Patricia Martin Tercero (Hosp. De La Marina Baixa), Esteban Martín Echevarria (Hosp. Universitario Guadalajara), María Martinez García (Hosp. General San Jorge), Rocío Martinez Gutierrez (Complejo Hosp. Universitario Asturias), Maribel Martinez Fernández (Complejo Hosp. Navarra), Caridad Martinez Torrealba (Complejo Hosp. Nuestra Señora Candelaria-Ofra), Ana Martínez García (Hosp. General Nuestra Señora Del Prado), Eva Martinez De Castro (Complejo Hosp. Universitario Marqués De Valdecilla), Rosario Martinez Barea (Complejo Hosp. Nuestra Señora De Valme), Vanessa Martínez Tormo (Hosp. General Universitario De Castellón), Begoña Martínez Carrasco (Hosp. General Nuestra Señora Del Prado), Antonio Jesús Martínez Ortega (Complejo Hosp. Torrecardenas), Juan Víctor Martos Vandussen (Complejo Hosp. Especialidades Virgen De La Victoria), Ana María Mata Martin (Complejo Hosp. Virgen Del Rocío), Francisco Mata Perdigon (Hosp. Universitario Puerta Del Mar), Isabel Mateo Gavira (Hosp. Universitario Puerta Del Mar), Virginia Mazoteras Muñoz (Hosp. General Universitario De Ciudad Real), Begoña Merelo Ruiz (Hosp. Infanta Elena Huelva), Almudena Milan Vegas (Hosp. Universitario Fundación Jimenez Diaz), Sarah Mills Gañan (Complejo Hosp. Universitario La Paz), Jose Pablo Miramontes Gonzalez (Hosp. Universitario Rio Hortega), Javier Modamio Molina (Hosp. Universitario Infanta Leonor), Laura Mola Reyes (Complejo Hosp. Universitario La Paz), Carmen Molina Villalba (Hosp. De Poniente), Juan Bautista Molina Soria (Hosp. San Agustin De Linares), Rosa Monfort García (Hosp. Universitario La Ribera), Jorge Monroy Sánchez (Hosp. Virgen De Las Nieves General), Nuria Pilar Montero Fernández (Complejo Hosp. Gregorio Marañón), Estefanía Montero Fernández (Hosp. Del Bierzo), Juan Antonio Montes Romero (Complejo Hosp. Torrecardenas), Juan Mora Delgado (Hosp. De Jerez), Damián Mora Peña (Hosp. Virgen De La Luz), Irene Moraleja Yudego (Hosp. Galdakao-Usansolo), María Luisa Morales Barroso (Hosp. De Jerez), Alicia Moreno Borreguero (Hosp. Universitario Fuenlabrada), Antonio Miguel Moreno García (Hosp. De Jerez), Miriam Moriana Hernández (Hosp. Clínico Universitario Valencia), Jose Mostazo Torres (Complejo Hosp. Regional Universitario Málaga), Rodrigo Muelas Soria (Hosp. General Universitario De Castellón), Araceli Muñoz Garach (Hosp. Virgen De Las Nieves General), Marta Muñoz Velez (Hosp. Comarcal El Escorial), Teo Muñoz De Escalona Martínez (Complejo Hosp. Torrecardenas), Gema Navarro Jiménez (Hosp. Universitario Infanta Sofia), María Jose Navarro Gonzalez (Hosp. Comarcal Noroeste De La Región De Murcia), Carmen Navarro Ceballos (Hosp. Universitario Severo Ochoa), Julio Nieto Ramirez (Complejo Hosp. Torrecardenas), Sonia Nieto Colino (Hosp. Universitario Móstoles), Mercedes Noval Font (Hosp. Universitario Son Espases), Cristina Novo Rodriguez (Hosp. Virgen De Las Nieves General), Luis Olay Gayoso (Complejo Hosp. Universitario Asturias), Génesis Estefanía Olaya Loor (Hosp. Universitario Rey Juan Carlos), Jose Gregorio Oliva García (Complejo Hosp. Nuestra Señora Candelaria-Ofra), Susana Olivera Gonzalez (Hosp. Marina Salud), Francisco Jesús Olmo Montes (Complejo Hosp. Virgen Macarena), Carolina Orduz Arenas (Complejo Hosp. Universitario Asturias), Jose María Ortega Morente (Hosp. Infanta Elena), Jose Jorge Ortez Toro (Hosp. Reina Sofia), Virginia Oses Zarate (Complejo Hosp. San Pedro La Rioja), Daniel Padron Guillen (Hosp. Universitario Clínico San Carlos), José Enrique Palacio Abizanda (Complejo Hosp. Nuestra Señora Candelaria-Ofra), Jose María Palacio Mures (Hosp. Universitario Rio Hortega), Fiorella Palmas Candia (Complejo Hosp. Vall D’Hebron), Vicente Palomar Abril (Hosp. Virgen De Los Lirios), Patricia Palomero Entrenas (Complejo Hosp. Cartagena), Carmen Pantin González (Hosp. Universitario Fuenlabrada), Armando Jose Pardo Gomez (Hosp. Universitario Puerta De Hierro Majadahonda), Teresa Pareja Sierra (Hosp. Universitario Guadalajara), Ginés David Parra García (Hosp. La Inmaculada), Rubén Pastor Mateu (Consorcio Hosp. General Universitario Valencia), Adrián Pastor Alcaraz (Complejo Hosp. Cartagena), Ruth Paz Maya (Hosp. Doctor Jose Molina Orosa), Noelia Peláez Torres (Hosp. Universitario Príncipe De Asturias), Lira Pelari (Hosp. Universitario Ramon Y Cajal), María José Penalva Moreno (Hosp. Universitario Infanta Sofia), Johanna Peña Vivas (Complejo Hosp. Universitario Asturias), Sonia Peña Balbuena (Complejo Asist. Zamora), Mariana Teresa Peña Perea (Complejo Hosp. Torrecardenas), Beatriz Perdomo Ramírez (Hosp. Universitario Fundación Alcorcón), Eva Perello Camacho (Hosp. Universitario Sant Joan D’Alacant), Nestor Fabricio Pereyra Venegas (Hosp. Doctor Jose Molina Orosa), Javier Perez Altozano (Hosp. Virgen De Los Lirios), Jose Perez Silvestre (Consorcio Hosp. General Universitario Valencia), María Del Mar Perez Martin (Complejo Hosp. Regional Universitario Málaga), Jose Luis Perez Aguiar (Complejo Hosp. Universitario Canarias), Carmen Perez Blanco (Hosp. Comarcal El Escorial), Patricia Perez Rodriguez (Hosp. Universitario Puerta De Hierro Majadahonda), Rocío Perez Abud (Hosp. Costa Del Sol), Miguel Ángel Perez Ramos (Hosp. Infanta Elena Huelva), Sergio Perez Pinto (Complejo Asist. Ávila), Ignacio Pérez Catalán (Hosp. General Universitario De Castellón), Luis Perez Belmonte (Complejo Hosp. Regional Universitario Málaga), Nieves Perez Climent (Hosp. Virgen De Los Lirios), Virginia Perez Vázquez (Hosp. Universitario Puerto Real), Nuria Pérez Martín (Complejo Hosp. Universitario Insular Materno Infantil), María Concepción Piñero Pérez (Complejo Hosp. Salamanca), Francisco Pita Gutierrez (Complejo Hosp. Universitario A Coruña), Ignacio Plata Perez (Hosp. General Básico Santa Ana), Eduardo Platero Rodrigo (Hosp. Universitario Guadalajara), Eduardo Polo Marques (Hosp. Royo Villanova), Ursula Ponce Villar (Hosp. Francesc De Borja), Jose Miguel Ponce Ortega (Complejo Hosp. Miguel Servet), Leonel Alejandro Porta González (Complejo Hosp. Toledo), Paloma Portillo Ortega (Hosp. General Universitario Reina Sofia), Jesus Portillo González (Complejo Hosp. Torrecardenas), Claudia Prada García (Complejo Asist. Universitario Burgos), Diana Prada Cotado (Hosp. Universitario De La Princesa), Cristina Prieto Prieto (Hosp. Universitario San Cecilio), Isabel Prieto Nieto (Complejo Hosp. Universitario La Paz), Lucía Prieto Coca (Hosp. Universitario Clínico San Carlos), David Primo Martin (Hosp. Clínico Universitario Valladolid), Laura Príncipe Mellado (Complejo Hosp. Universitario 12 De Octubre), Fran Puchades Gimeno (Consorcio Hosp. General Universitario Valencia), Joaquín Puerma Ruiz (Hosp. Universitario Del Sureste), Begoña Quintana Ángel (Complejo Hosp. Virgen Del Rocío), Sara Quintana Arroyo (Complejo Hosp. Universitario Insular Materno Infantil), Karina Liz Quiñones Huayna (Hosp. Universitario Severo Ochoa), Juan Quirós Rivero (Complejo Hosp. Universitario Badajoz), Julio Ramírez Luna (Hosp. Virgen De La Luz), Mayte Ramiz Martinez (Hosp. Universitario Basurto), Fátima Ramón Vigo (Complejo Hosp. Jaén), Araceli Ramos Carrasco (Hosp. Universitario Móstoles), Esther Ramos Muñoz (Hosp. Universitario Clínico San Carlos), Antonio J Ramos Guerrero (Complejo Hosp. Virgen Del Rocío), Juan Ignacio Ramos Clemente Romero (Hosp. Infanta Elena Huelva), Reyes Ravé García (Complejo Hosp. Virgen Macarena), Ángel Rebollo Román (Complejo Hosp. Reina Sofia), Laura Redondo Robles (Complejo Hosp. Salamanca), Idoia Reta Decoreau (Hosp. Universitario Basurto), Laura Rey Fernández (Hosp. Costa Del Sol), María Riestra Fernández (Hosp. Cabueñes), Isabel Mª Ríos Holgado (Complejo Asist. Campo De Gibraltar), Alejandra Maricel Rivas Montero (Complejo Hosp. Gregorio Marañón), Rosmeri Rivera Irigoin (Fund. Hosp. Son Llatzer), Francisco Robles Agudo (Complejo Hosp. Universitario La Paz), Francisco Rodriguez Diaz (Hosp. Comarcal De La Axarquia), María Amparo Rodriguez Piñera (Hosp. Cabueñes), Jaime Rodriguez Salazar (Hosp. Universitario Severo Ochoa), Orvelindo Rodriguez Hernández (Complejo Hosp. Universitario Canarias), Ángel Rodriguez Sanchez (Hosp. Universitario Basurto), Laura Rodriguez Pausin (Complejo Hosp. Universitario Canarias), Gonzalo Rodriguez Chimeno (Complejo Hosp. Universitario Canarias), Juan Pedro Rodríguez Rodríguez (Hosp. La Inmaculada), Nazareth Rodríguez Novo (Hosp. Universitario Lucus Augusti), Jesús Manuel Rodríguez De León (Complejo Hosp. Universitario Insular Materno Infantil), Pablo Rodríguez De Vera Gómez (Complejo Hosp. Virgen Macarena), Celia Roig Marti (Hosp. General Universitario De Castellón), Belen Roldan García (Complejo Hosp. Universitario Albacete), Alejandro Rolo Ramirez (Complejo Hosp. Salamanca), Esperanza Romero Rodríguez (Complejo Asist. Campo De Gibraltar), Daniel Romero Esteban (Hosp. Clínico Universitario Virgen De La Arrixaca), Roberto Carlos Romo Remigio (Complejo Hosp. Jaén), Arantxa Rubio Marcos (Hosp. Clínico Universitario Valencia), Jaime Rubio Perez (Hosp. Universitario Fundación Jimenez Diaz), Patricia Rubio Marín (Hosp. De Jerez), Montserrat Rueda Sanchez (Hosp. General Universitario De Ciudad Real), María Visitación Ruiz García (Complejo Hosp. Torrecardenas), María Ruiz Ruigomez (Complejo Hosp. Universitario 12 De Octubre), Almudena Ruiz Molina (Hosp. Galdakao-Usansolo), Rocío Ruiz Hueso (Complejo Hosp. Virgen Macarena), Mario Salas Carrillo (Hosp. Universitario Clínico San Carlos), Juan Diego Salazar Leon (Hosp. Universitario Doctor Peset), Teresa Salcedo Peris (Hosp. General Universitario De Ciudad Real), Renato Darío Salguero Aguilar (Complejo Hosp. Torrecardenas), Lourdes Salinero Gonzalez (Hosp. General Universitario Reina Sofia), Sergio Salmerón Ríos (Complejo Hosp. Universitario Albacete), Christian Salom Vendrell (Hosp. Universitario Doctor Peset), Carlos Salvador Suarez (Hosp. La Plana), María Isabel Salvador Perez (Hosp. Universitario La Ribera), Miguel Sampedro Nuñez (Hosp. Universitario De La Princesa), Emilio Sanchez Navarro (Hosp. General Universitario Morales Meseguer), Carlos Sanchez Juan (Consorcio Hosp. General Universitario Valencia), Francisco Jose Sanchez Torralvo (Complejo Hosp. Regional Universitario Málaga), Ángeles Sánchez Gálvez (Complejo Hosp. Jaén), Nancy María Sanchez Gómez Hosp. De Jerez), Dario Sanchez Cabrero (Complejo Hosp. Universitario La Paz), Ángel Sanchez Iglesias (Hosp. General Universitario De Castellón), Olivia Sánchez Sánchez (Hosp. Universitario Ramon Y Cajal), Macarena Sanchez-Gallego Alonso (Complejo Hosp. Universitario Canarias), Myriam Sanchez-Pacheco Tardon (Hosp. General Universitari D’Alacant), Olga Margarita Sanchez-Vilar Burdiel (Hosp. Universitario Fundación Jimenez Diaz), Paula Santos Patiño (Complejo Hosp. Toledo), Alejandro Sanz Paris (Complejo Hosp. Miguel Servet), Clara Serrano Moreno (Complejo Hosp. Gregorio Marañón), Maryam Sidahi Serrano (Hosp. Infanta Elena Huelva), Mariela Pia Silva Pomarino (Hosp. General Universitario De Castellón), Paula Simón Silva (Complejo Hosp. Universitario Badajoz), Jorge Soler Lopez (Hosp. General Universitario De Castellón), Isaac Solero Martin (Complejo Hosp. La Fe), Pilar Sorando Fernández (Hosp. Universitario Basurto), Francisco Soria Perdomo (Complejo Hosp. Universitario 12 De Octubre), Jesús Soto Benítez (Hosp. Universitario Puerta Del Mar), Francisco Manuel Suarez García (Hosp. San Agustin), Lorena Suarez Gutierrez (Complejo Hosp. Universitario Asturias), Ana Suero Roiz (Hosp. Universitario Puerta De Hierro Majadahonda), María Talaya Alarcón (Hosp. Universitario De La Princesa), Luis Tallón Aguilar (Complejo Hosp. Virgen Del Rocío), María Jose Tapia Guerrero (Complejo Hosp. Regional Universitario Málaga), Carlos Taxonera Samso (Hosp. Universitario Clínico San Carlos), Alicia Cristina Tejera Concepcion (Complejo Hosp. Nuestra Señora Candelaria-Ofra), Pedro Tomás Tobaruela (Hosp. Universitario Guadalajara), Sara Torrejón Jaramillo (Hosp. De Sant Joan Despi Moises Broggi), Emiliano Torres García (Hosp. Virgen Del Puerto), Beatriz Torres Torres (Hosp. Clínico Universitario Valladolid), María Del Castillo Tous Romero (Complejo Hosp. Virgen Macarena), Isabel Tovar Martín (Hosp. Virgen De Las Nieves General), Pablo Trincado Aznar (Complejo Hosp. Miguel Servet), Vanessa Triviño Yannuzzi (Hosp. Universitario Infanta Leonor), Virginia Urquijo Mateos (Hosp. Universitario Cruces), Leandro Valdes Disla (Hosp. Universitario Fundación Alcorcón), Sara Valle Rodriguez- Navas (Hosp. Galdakao-Usansolo), Alvaro Valverde Márquez (Complejo Hosp. Salamanca), Pilar Varela Trastoy (Hosp. Cabueñes), Mercedes Vázquez Gutiérrez (Complejo Hosp. Torrecardenas), Juan Antonio Vázquez Rodríguez (Hosp. De Poniente), Carlos Mauricio Vega Aponte (Hosp. Universitario Severo Ochoa), Christian Velardo Andres (Hosp. Virgen Del Puerto), Jara Velasco Garcia-Cuevas (Hosp. Comarcal El Escorial), Enmanuel Velasquez Zambrano (Hosp. Costa Del Sol), Laura Velázquez Ríos (Hosp. Universitario Fuenlabrada), Elena Vera Tuñon (Complejo Hosp. La Fe), Victoria Vera Barragan (Complejo Hosp. Universitario Badajoz), Guayente Verdes Sanz (Hosp. Alcañiz), Lorena Verguizas Gallego (Hosp. Universitario Infanta Leonor), Silvia Veses Martin (Hosp. Universitario Doctor Peset), Sofia Vidal Serrano (Complejo Hosp. Virgen Del Rocío), Concepcion Vidal Peracho (Hosp. Royo Villanova), Alejandro Viejo Almanzor (Hosp. Universitario Puerta Del Mar), Jose Carlos Villa Guzman (Hosp. General Universitario De Ciudad Real), Javier Villanueva Martinez (Hosp. Universitario Fundación Alcorcón), Marta Villarino Sanz (Hosp. Universitario Infanta Sofia), Francisco Villazon Gonzalez (Complejo Hosp. Universitario Asturias), Cristina Viñolo Ubiña (Hosp. De Poniente), Lucia Visiedo Rodas (Hosp. Costa Del Sol), Ximena Carolina Vivas Vaca (Complejo Hosp. Salamanca), Esteban Alessandro Vogt Sánchez (Complejo Hosp. Torrecardenas), Jaime Wong Cruz (Hosp. Reina Sofia), Ning Yun Wu Xiong (Hosp. Clínico Universitario Valencia), Carmen Yeste Doblas (Hosp. Universitario San Cecilio), Manuel Zalabardo Aguilar (Complejo Hosp. Especialidades Virgen De La Victoria), Yolanda Zambrano Huerta (Hosp. Doctor Jose Molina Orosa), Eduardo Zamorano Gonzalez (Complejo Hosp. Especialidades Virgen De La Victoria), Gabriela Zapata Maldonado (Complejo Hosp. Universitario Marqués De Valdecilla), Jose Zapatero Ortuño (Hosp. Universitario Rey Juan Carlos), Cristina Zaragoza Brehcist (Complejo Hosp. Universitario 12 De Octubre).
