# Supplementary material for: Physicians’ Perception of Oral Nutritional Supplement Acceptance and Tolerability in Malnourished Outpatients: PerceptiONS Study
Source: Nutrients. 2023 Feb 28;15(5):1219. doi: 10.3390/nu15051219 (PMC10005370; doi:10.3390/nu15051219)
Supplement: Supplementary file 1 [file nutrients-15-01219-s001.zip › nutrients-2225504-supplementary.pdf]

Table S1. Patients' sociodemographic characteristics

Patients' characteristics	% of patients
Age, category, years, %	
≤ 45 years	7.11%
46 to 55 years	10.57%
56 to 65 years	18.84%
66 to 75 years	25.79%
> 75 years	37.68%
Sex, %	
Male, %	53.58%
Female, %	46.42%
Time in treatment, category, days, %	
30-59 days, %	4.01%
60-120 days, %	90.02%
121-199 days, %	5.96%
The tool employed to evaluate malnutrition, %*	
GLIM, %	30.88%
MUST, %	22.69%
MNA, %	20.23%
Physician's subjective criteria, %	19.12%
SGA, %	8.43%
CONUT, %	7.79%
NRS-2002, %	4.29%
MST, %	3.86%
Unspecified, %	2.54%
Pathology justifying treatment with ONS, %*	
Cancerous cachexia due to chronic enteritis caused by chemo- and/or radiotherapy treatment, %	5.17%
Inflammatory bowel disease, %	8.43%
Oncologic patient undergoing active chemotherapy or radiotherapy treatment, %	14.79%
Convalescent pluripathological patient, %	17.13%
Non-tumoral esophageal stricture, %	1.11%
Cystic fibrosis, %	0.32%
Low debit enterocutaneous fistulas, %	0.72%
Hip fracture, %	6.68%
Chronic renal insufficiency in adults, %	3.86%
Patient with severe heart disease, %	5.68%
Malnourished patient undergoing scheduled major surgery or transplantation, %	5.88%
Severe COPD patient, %	5.29%
	2.07%

Infectious medical pathology involving severe malabsorption, %	5.33%
Severe malabsorption syndrome, %	1.87%
Severe intestinal motility disorder, %	
Head and neck tumor/ENT/Maxillofacial Surgery, %	11.72%
Brain tumor, %	0.99%
Digestive tumor, %	18.20%
Other	10.06%

*Physicians could choose more than one answer. Abbreviations: CONUT: Controlling Nutritional Status; GLIM: Global Leadership Initiative on Malnutrition; MST: Malnutrition Screening Tool; MUST: Malnutrition Universal Screening Tool. NMA: Mini Nutritional Assessment; NRS-2002: Nutrition Risk Screening 2002; SGA: Subjective global assessment.

Table S2. Complete questionnaire

**Physicians' perspective on patient adherence to ONS**			
Question	Answer		
1. From your perspective, what has been the degree of adherence to the prescribed treatment? Please select the answer that best fits the patient.	1. Non-adherence to prescribed treatment<br>2. < 25% of prescribed treatment<br>3. 25 to 50% of prescribed treatment<br>4. 51 to 75% of prescribed treatment<br>5. >75% of prescribed treatment		
2.a) Of the following organoleptic properties (smell, flavor, texture), which most influenced adherence to the nutritional supplement?<br>b) Which of the following 3 options (organoleptic properties, the number of daily intakes prescribed, consumption of the supplement maintained over time) most positively influenced patient adherence?	Rank in order of importance (1 = least important; 3 = most important)		
**Physicians' perspective on patient acceptance/satisfaction with ONS**			
Question	Answer		
3. From your point of view, is the patient satisfied with the ONS received?	1. not at all<br>2. a little<br>3. quite a lot<br>4. a lot		
4. From your point of view, is the patient satisfied with the ONS's organoleptic properties (smell, taste, texture)?	Smell<br>1. not at all<br>2. a little<br>3. quite a lot<br>4. a lot	Taste<br>1. not at all<br>2. a little<br>3. quite a lot<br>4. a lot	Texture<br>1. not at all<br>2. a little<br>3. quite a lot<br>4. a lot
5. From your perspective, do you think the ONS organoleptic properties (smell, taste, texture) have influenced patient satisfaction?	1. not at all<br>2. a little<br>3. quite a lot<br>4. a lot		

6. From your point of view, what is the patient's degree of acceptance of ONS in their daily diet?	1. not at all<br>2. a little<br>3. quite a lot<br>4. a lot
7. From your point of view, do you think the patient is satisfied with the benefit of the ONS?	1. not at all<br>2. a little<br>3. quite a lot<br>4. a lot
**Physicians' perspective on patient clinical improvement after taking the ONS**	
Question	Answer
8. Indicate, from your perspective, how much ONS have contributed to:<br>a) Improving the patient's general condition<br>b) Improving the patient's mood<br>c) Improving the patient's vitality/energy<br>d) Improving the patient's autonomy/functionality<br>e) Interfering with the patient's daily intake<br>f) Achieving weight gain in the patient<br>g) Improving the patient's quality of life	1. not at all<br>2. a little<br>3. quite a lot<br>4. a lot
9. Specific question for each ONS<br>a) Hypercaloric, high-protein peptide ONS rich in medium chain triglycerides (MCTs) without fiber. From your perspective, has the patient's gastrointestinal discomfort improved with the ONS? From your perspective, what symptoms (diarrhea, nausea, vomiting, abdominal pain, bloating) have improved?<br>b) Hypercaloric, high-protein ONS enriched with HMB and fructooligosaccharides (FOSs) From your point of view, has the ONS improved the patient's physical condition?<br>c) Hypercaloric, high-protein diabetes-specific ONS with high monounsaturated fatty acids (MUFAs). From your perspective,	1. not at all<br>2. a little<br>3. quite a lot<br>4. a lot

has taking ONS contributed to patient's better glycemic control?	
**Physician satisfaction**	
Question	Answer
10. After your experience, would you prescribe the same ONS again?	1. Yes<br>2. No

Table S3. Physicians' perspective on patient's clinical improvement after taking the ONS

Question 8. Indicate, from your perspective, how much has ONS contributed to:	
Improving the patient's general condition, %	87.04%
Improving the patient's quality of life, %	81.96%
Improving the patient's vitality/energy, %	81.28%
Improving the patient's autonomy/functionality, %	74.36%
Improving the patient's mood, %	66.85%
Achieving weight gain in the patient, %	64.47%
Interfering with the patient's daily intake, %	19.28%

Table S4. Subgroups analyses

1) Survey results for each ONS. Please, note that:

ONS A) is a hypercaloric, high-protein peptide ONS rich in MCTs without fiber

ONS B) is a hypercaloric, high-protein ONS enriched with HMB and FOS

ONS C) is a hypercaloric, high-protein ONS specific for diabetes with high MUFAs

**Physicians' perspective on patient adherence to ONS**				
1. From your perspective, what has been the degree of adherence to the prescribed treatment? Please select the answer that best fits the patient				
N=2516, %	Total	ONS A	ONS B	ONS C
Non-adherence to prescribed treatment	0.87%	1.18%	0.86%	0.63%
< 25% of prescribed treatment	3.70%	3.39%	3.92%	3.66%
25 to 50% of prescribed treatment	9.06%	8.98%	9.47%	8.59%
51 to 75% of prescribed treatment	29.25%	31.22%	27.56%	29.80%
>75% of prescribed treatment	57.11%	55.23%	58.18%	57.32%
2. a) Of the following organoleptic properties (smell, flavor, texture), which most influenced adherence to the nutritional supplement? Rank in order of importance (1 = least important; 3 = most important)				
N=2516, %	Total	ONS A	ONS B	ONS C
1st				

Smell<br>Flavor<br>Texture	43.72%<br>38.71%<br>17.57%	43.74%<br>38.59%<br>17.67%	44.59%<br>38.66%<br>16.75%	42.55%<br>38.89%<br>18.56%
2nd<br>Smell<br>Flavor<br>Texture	 34.38%<br>37.12%<br>28.50%	 35.79%<br>40.06%<br>24.15%	 33.78%<br>37.13%<br>29.09%	 33.96%<br>34.60%<br>31.44%
3rd<br>Smell<br>Flavor<br>Texture	 21.90%<br>24.17%<br>53.93%	 20.47%<br>21.35%<br>58.17%	 21.63%<br>24.21%<br>54.16%	 23.48%<br>26.52%<br>50.00%
b) Which of the following 3 options (organoleptic properties, the number of daily intakes prescribed, consumption of the supplement maintained over time) most positively influenced patient adherence? Rank in order of importance (1 = least important; 3 = most important)				
N=2516, %	Total	ONS A	ONS B	ONS C
1st<br>Organoleptic properties<br>The number of daily intakes prescribed<br>Consumption of the supplement maintained over time	 61.05%<br>18.28%<br>20.67%	 67.16%<br>13.55%<br>19.29%	 58.95%<br>20.48%<br>20.57%	 58.59%<br>19.44%<br>21.97%
2nd<br>Organoleptic properties<br>The number of daily intakes prescribed<br>Consumption of the supplement maintained over time	 18.24%<br>60.14%<br>21.62%	 13.99%<br>63.48%<br>22.53%	 21.44%<br>58.18%<br>20.38%	 17.68%<br>59.85%<br>22.47%
3rd<br>Organoleptic properties<br>The number of daily intakes prescribed<br>Consumption of the supplement maintained over time	 20.71%<br>21.58%<br>57.71%	 18.85%<br>22.97%<br>58.17%	 19.62%<br>21.34%<br>59.04%	 23.74%<br>20.71%<br>55.56%
**Physicians' perspective on patient's acceptance/satisfaction with ONS**				
3. From your point of view, is the patient satisfied with ONS received?				
N=2516, %	Total	ONS A	ONS B	ONS C
Not at all/a little	9.90%	10.60%	10.72%	8.21%
Quite a lot/a lot	90.10%	89.40%	89.28%	91.79%
4. From your point of view, is the patient satisfied with the ONS's organoleptic properties (smell, taste, texture)?				
N=2516, %	Total	ONS A	ONS B	ONS C
Smell				
Not at all/a little	18.84%	20.03%	19.23%	17.30%
Quite a lot/a lot	81.16%	79.97%	80.77%	82.70%
Taste				
Not at all/a little	11.84%	15.17%	11.10%	9.97%
Quite a lot/a lot	88.16%	84.83%	88.90%	90.03%
Texture				

Not at all/a little	15.14%	14.87%	15.69%	14.65%
Quite a lot/a lot	84.86%	85.13%	84.31%	85.35%
5. From your perspective, do you think the ONS organoleptic properties (smell, taste, texture) have influenced patient satisfaction?				
N=2516, %	Total	ONS A	ONS B	ONS C
Not at all/a little	9.58%	10.60%	9.76%	8.46%
Quite a lot/a lot	90.42%	89.4%	90.24%	91.54%
6. From your point of view, what is the patient's degree of acceptance of ONS in their daily diet?				
N=2516, %	Total	ONS A	ONS B	ONS C
Not at all/a little	11.37%	12.52%	11.96%	9.60%
Quite a lot/a lot	88.63%	87.48%	88.04%	90.40%
7. From your point of view, do you think the patient is satisfied with the benefit of the ONS?				
N=2516, %	Total	ONS A	ONS B	ONS C
Not at all/a little	11.49%	12.08%	12.25%	9.97%
Quite a lot/a lot	88.51%	87.92%	87.75%	90.03%
**Physicians' perspective on patient clinical improvement after taking the ONS**				
8. Indicate, from your perspective, how much ONS have contributed to:				
N=2516, %	Total	ONS A	ONS B	ONS C
a) Improving the patient's general condition				
Not at all/a little	12.96%	13.11%	13.59%	11.99%
Quite a lot/a lot	87.04%	86.89%	86.41%	88.01%
b) Improving the patient's mood				
Not at all/a little	33.15%	32.99%	34.83%	31.06%
Quite a lot/a lot	66.85%	67.01%	65.17%	68.94%
c) Improving the patient's vitality/energy				
Not at all/a little	18.72%	19.44%	17.70%	19.44%
Quite a lot/a lot	81.28%	80.56%	82.30%	80.56%
d) Improving the patient's autonomy/functionality				
Not at all/a little	25.64%	26.07%	24.78%	26.39%
Quite a lot/a lot	74.36%	73.93%	75.22%	73.61%
e) Interfering with the patient's daily intake				
Not at all/a little	80.72%	78.65%	81.91%	80.93%
Quite a lot/a lot	19.28%	21.35%	18.09%	19.07%
f) Achieving weight gain in the patient				
Not at all/a little	35.5%	32.99%	38.76%	33.46%
Quite a lot/a lot	64.47%	67.01%	61.24%	66.54%
g) Improving the patient's quality of life				
Not at all/a little	18.04%	17.67%	18.56%	17.68%
Quite a lot/a lot	81.96%	82.33%	81.44%	82.32%
9. Specific question for each ONS				
a) Hypercaloric, high-protein peptide ONS rich in medium chain triglycerides (MCTs) without fiber.				

From your perspective, has the patient's gastrointestinal discomfort improved with the ONS? From your perspective, what symptoms (diarrhea, nausea, vomiting, abdominal pain, bloating) have improved?				
N=638, %	Total	ONS A	ONS B	ONS C
Not at all/a little	----	28.53%	----	----
Quite a lot/a lot	----	71.47%	----	----
Diarrhea				
Not at all/a little	----	21.16%	----	----
Quite a lot/a lot	----	59.09%	----	----
Nausea				
Not at all/a little	----	25.39%	----	----
Quite a lot/a lot	----	35.74%	----	----
Vomiting				
Not at all/a little	----	22.41%	----	----
Quite a lot/a lot	----	27.27%	----	----
Abdominal pain				
Not at all/a little	----	26.18%	----	----
Quite a lot/a lot	----	51.72%	----	----
Bloating				
Not at all/a little	----	22.88%	----	----
Quite a lot/a lot	----	55.33%	----	----
b) Hypercaloric, high-protein ONS enriched with HMB and fructooligosaccharides (FOSs) From your point of view, has the ONS improved the patient's physical condition?				
N=863, %	Total	ONS A	ONS B	ONS C
Not at all/a little	----	----	17.84%	----
Quite a lot/a lot	----	----	82.16%	----
c) Hypercaloric, high-protein diabetes-specific ONS with high monounsaturated fatty acids (MUFAs). From your perspective, has taking ONS contributed to patient's better glycemic control?				
N=749, %	Total	ONS A	ONS B	ONS C
Not at all/a little	----	----	----	24.43%
Quite a lot/a lot	----	----	----	75.57%

2) Three questions of interest were studied in the subgroup analysis by patient's age (≤ 65 and > 65 years)

**Physicians' perspective on patient clinical improvement after taking the ONS**		
From your perspective, how much has ONS contributed to improving the patient's autonomy/functionality?		
	≤ 65 años (N=919)	> 65 años (N=1597)
Not at all/a little	75.30%	73.83%
Quite a lot/a lot	24.70%	26.17%

From your perspective, how much has ONS contributed to achieving weight gain in the patient?		
	≤ 65 años (N=919)	> 65 años (N=1597)
Not at all/a little	68.34%	62.24%
Quite a lot/a lot	31.66%	37.76%
**Physicians' perspective on patient adherence to ONS**		
From your perspective, what has been the degree of adherence to the prescribed treatment? Please select the answer that best fits the patient		
	≤ 65 años (N=919)	> 65 años (N=1597)
Non-adherence to prescribed treatment	1.20%	0.69%
< 25% of prescribed treatment	3.70%	3.69%
25 to 50% of prescribed treatment	9.58%	8.77%
51 to 75% of prescribed treatment	27.75%	30.12%
>75% of prescribed treatment	57.78%	56.73%

3) Four questions of interest were studied in the subgroup analysis by underlying pathology (oncology and non-oncology patients)

**Physicians' perspective on patient clinical improvement after taking the ONS**		
From your perspective, how much has ONS contributed to interfere with the patient's daily intake?		
	oncology (N=1121)	non-oncology (N=1395)
Not at all/a little	80.64%	80.79%
Quite a lot/a lot	19.36%	19.21%
**Physicians' perspective on patient acceptance/satisfaction with ONS**		
From your point of view, is the patient satisfied with the ONS taste?		
	oncology (N=1121)	non-oncology (N=1395)
Not at all/a little	13.20%	10.75%
Quite a lot/a lot	86.80%	89.25%
From your point of view, what is the patient's degree of acceptance of ONS in their daily diet?		
	oncology (N=1121)	non-oncology (N=1395)
Not at all/a little	11.69%	11.11%
Quite a lot/a lot	88.31%	89.89%
**Physicians' perspective on patient adherence to ONS**		
From your perspective, what has been the degree of adherence to the prescribed treatment? Please select the answer that best fits the patient		
	oncology (N=1121)	non-oncology (N=1395)
Non-adherence to prescribed treatment	0.62%	1.08%
< 25% of prescribed treatment	4.01%	3.44%

25 to 50% of prescribed treatment	9.63%	8.60%
51 to 75% of prescribed treatment	29.26%	29.25%
>75% of prescribed treatment	56.47%	57.63%
